# Association between HIF1A P582S and A588T Polymorphisms and the Risk of Urinary Cancers: A Meta-Analysis

**DOI:** 10.1371/journal.pone.0063445

**Published:** 2013-05-27

**Authors:** Dawei Li, Jikai Liu, Wenhua Zhang, Juchao Ren, Lei Yan, Hainan Liu, Zhonghua Xu

**Affiliations:** 1 Department of Urology, Qilu Hospital of Shandong University, Shandong, China; 2 Key Laboratory of Cardiovascular Remodeling and Function Research Affiliated to Ministry of Education of P.R.China and Ministry of Public Health of P.R.China, Jinan, P.R.China; University of Aberdeen, United Kingdom

## Abstract

**Purpose:**

The hypoxia-inducible factor-1 alpha (HIF1A) plays a vital role in cancer initiation and progression. Previous studies have reported the existence of HIF1A P582S and A588T missense polymorphisms in renal, urothelial and prostatic carcinomas, however the effects remain conflicting. Therefore, we performed a meta-analysis to assess the association between these sites and the susceptibility of urinary cancers.

**Methods:**

We searched the PubMed database without limits on language until Nov 25, 2012 for studies exploring the relationship of HIF1A P582S and A588T polymorphisms and urinary cancers. Still, article search was supplemented by screening the references of retrieved studies manually. Odds ratios (OR) and 95% confidence intervals (95% CI) were calculated to evaluate the strength of the associations between the two by RevMan 5.0 software. Simultaneously, publication bias was estimated by funnel plot and Begg’s test with Stata 12.1 software.

**Results:**

Overall, 11 individual case-control studies with 5195 cases and 5786 controls for P582S polymorphism, and 9 studies with 3482 cases and 4304 controls for A588T polymorphism were respectively included in the final meta-analysis. For HIF1A P582S polymorphism, individuals with TT genotype showed 1.60 fold higher risk than the others carrying CT or CC genotypes in Caucasian population (OR = 1.60, 95% CI = 1.09–2.33, *P*_heterogeneity_ = 0.11, *P* = 0.02). For HIF1A A588T polymorphism, the A allele was significantly correlated with higher urinary cancers risk in Asian population (OR = 1.41, 95% CI = 1.03–1.93, *P*_heterogeneity_ = 0.22, *P* = 0.03). Still, significant associations were found for prostate cancer in the allele and dominant models (OR = 1.46, 95% CI = 1.01–2.12, *P*_heterogeneity_ = 0.49, *P* = 0.04 and OR = 1.45, 95% CI = 1.00–2.12, *P*_heterogeneity_ = 0.50, *P* = 0.05).

**Conclusions:**

The current findings suggest that HIF1A P582S polymorphism correlates with urinary cancers risk in Caucasian population, while A588T polymorphism may increase the risk of urinary cancers in Asian population and prostate cancer.

## Introduction

Cancer, known as a malignant neoplasm, is involving in unregulated cell growth. Approximately 12.7 million cancers were newly diagnosed and 7.6 million people died of cancer worldwide [1]. Renal cell cancer, urothelial carcinoma and prostate cancer are common types of malignancies worldwide [1]. Up to now, the exact mechanisms of carcinogenesis have not yet been fully elucidated. It is essential to explore the potential genetic and protein markers for screening, early diagnosing and predicting the occurrence as well as prognosis for urinary cancers.

Hypoxia refers to low oxygen condition and is common in solid tumors [2]. The protein encoded by the hypoxia-inducible factor-1 alpha (HIF1A) gene is a key transcription factor found in cells growing at low oxygen concentrations, which regulates cellular responses, adaption and survival under hypoxia in physiology and pathological processes [3], [4] via the increased transcription of several dozens of target genes (VEGF [5], DDX3 [6], iNOS [7], CX3CR1 [8], etc.). Thus, both HIF1A and its encoding gene are supposed to be promising candidates in the pathogenesis of cancers [9]. Human HIF1A gene locates at chromosome 14q21–24 [10], composes of 15 exons, codes the cDNA of 3919 bps, and produces the protein of 826 amino acids. Single nucleotide polymorphisms (SNP) in coding regions sometimes result in amino acid substitutions and affect the functional properties of translated protein. Two most widely studied missense polymorphisms, P582S (Pro582Ser, C1772T, rs11549465) and A588T (Ala588Thr, G1790A, rs11549467), have been detected within the oxygen-dependent degradation (ODD) domain in exon 12 of the gene. A base change from C to T at 1772 leads rise to Pro/Ser variation at codon 582, while the base alteration from G to A at 1790 gives rise to Ala/Thr variation at codon 588 [11]. It is therefore of added significance to identify genetic defects of HIF1A gene responsible for its enzyme activity. The HIF1A genetic P582S and A588T polymorphisms have been supposed to be accountable for the risk of urinary cancers. However, the results from epidemiological studies have been controversial and inconsistent [11]–[21]. The case-control study carried out by Ollerenshaw M [21] *et al* found that HIF1A P582S and A588T polymorphisms would confer susceptibility to RCC. Still, three additional studies by Foley R [14], Chau CH [20] and Orr-Urtreger A [19] *et al* demonstrated that men with HIF1A P582S polymorphism had a higher risk of prostate cancer. Li P [12] *et al* reported that HIF1A A588T rather than P582S polymorphism contributed to increased risk of prostate caner. In contrast, Nadaoka J [17] and Qin C [13] *et al* showed the data that these polymorphisms correlated closely with the progression of transitional cell carcinoma (TCC) and renal cell carcinoma (RCC) but the onset of TCC and RCC. Jacobs EJ [16] *et al* even indicated that the rate of A588T polymorphism was lower in prostate cancer patients. Meanwhile, several studies done by other groups [11], [15], [18] failed to detect any association between HIF1A P582S and A588T polymorphisms and the risk of urinary cancers.

The inconsistent conclusions may have resulted from differences in patient ethnic backgrounds and relatively small sample sizes. In this study, we collected and summarized published case-control studies on the two most widely studied polymorphisms in urinary cancers to shed light on current uncertain claims.

## Materials and Methods

### Identification and Inclusion of Studies

In current meta-analysis, the database of PubMed was scrutinized without limits on language until Nov 25^th^, 2012. Epidemiologic studies exploring the relationship of HIF1A P582S and/or A588T polymorphisms and urinary cancers were identified. The following keywords were adopted: (hypoxia-inducible factor-1 OR hypoxia-inducible factor OR HIF-1 OR HIF1A OR HIF) and (polymorphism OR variant OR SNP OR mutation) and (kidney OR renal OR urothelial OR transitional cell carcinoma OR bladder OR prostatic OR prostate). Meanwhile, the references of eligible studies were manually screened for potential case-control studies. Finally, a total of 248 abstracts meeting the search criteria were retrieved. The eligibility criteria of the meta-analysis were: (a) The studies had to be case-control studies exploring the associations between HIF1A P582S and/or A588T polymorphisms and urinary cancers; (b) The studies provided the number of cases and controls for various genotypes. The exclusion criteria of the meta-analysis were: (a) animal studies; (b) reviews, editorial, comments; (c) studies with duplicate data. On screening titles, abstracts and full texts, 11 eligible studies conformed to inclusion criteria were finally included.

### Data Collection

For each study, we extracted data through a standard form. The following characteristics were respectively extracted from the included studies: name of first author, year of publications, country of origin, ethnicity, gender of recruited subjects, cancer types, numbers of various genotypes in case and control groups, methods for detecting HIF1A P582S and/or A588T polymorphisms, Hardy-Weinberg equilibrium (HWE). In the case of disagreement, discrepancies of included studies were resolved by discussion.

### Statistical Methods

The genotypes and alleles difference of HIF1A P582S and A588T polymorphisms in Caucasian and Asian populations was calculated by chi-square test. HWE for HIF1A P582S and A588T polymorphisms of control groups was extracted from the original studies. In case of studies without reporting HWE status, HWE in control group was calculated by the chi-square test. And a *P*-value less than 0.05 was considered to be statistically significant. We evaluated the contribution of HIF1A P582S and A588T polymorphisms to the risk of urinary cancers by adopting the RevMan software 5.0, which is developed by Cochrane Collaboration. For HIF1A P582S polymorphism, we evaluated the risk in the dominant model (TT+CT vs. CC), the recessive model (TT vs. CT+CC) and the allele model (T vs. C) respectively. For HIF1A A588T polymorphism, we only evaluated the risk in the dominant model (AA+AG vs. GG) and the allele model (A vs. G) due to few frequencies of genotype AA in subjects. Then, we performed subgroup meta-analysis according to the status of HWE, cancer type and ethnicity. The strength of association was estimated by calculating ORs and the corresponding 95% CIs. Still, a *P*-value less than 0.05 was considered to be statistically significant. Heterogeneity assumption was assessed by the chi-square based Q test and was regarded to be statistically significant if *P*<0.10. The random-effects model (the Dersimonian-Laird method) would be used if the test of heterogeneity was significant; otherwise the fixed-effects model (the Mantel-Haenszel method) would be applied in the analysis [22], [23]. Sensitivity analyses were carried out to assess the stability of the final results by conducting subgroup meta-analysis of studies with controls in HWE. The potential publication bias was primarily appraised by the funnel plot. An asymmetric plot suggests a possible publication bias. Funnel plot asymmetry was further evalued by Begg’s [24] test with STATA 12.1 software. A *P*-value less than 0.05 was considered to be statistically significant.

## Results

### Characteristics of Included Studies

**Figure 1:**
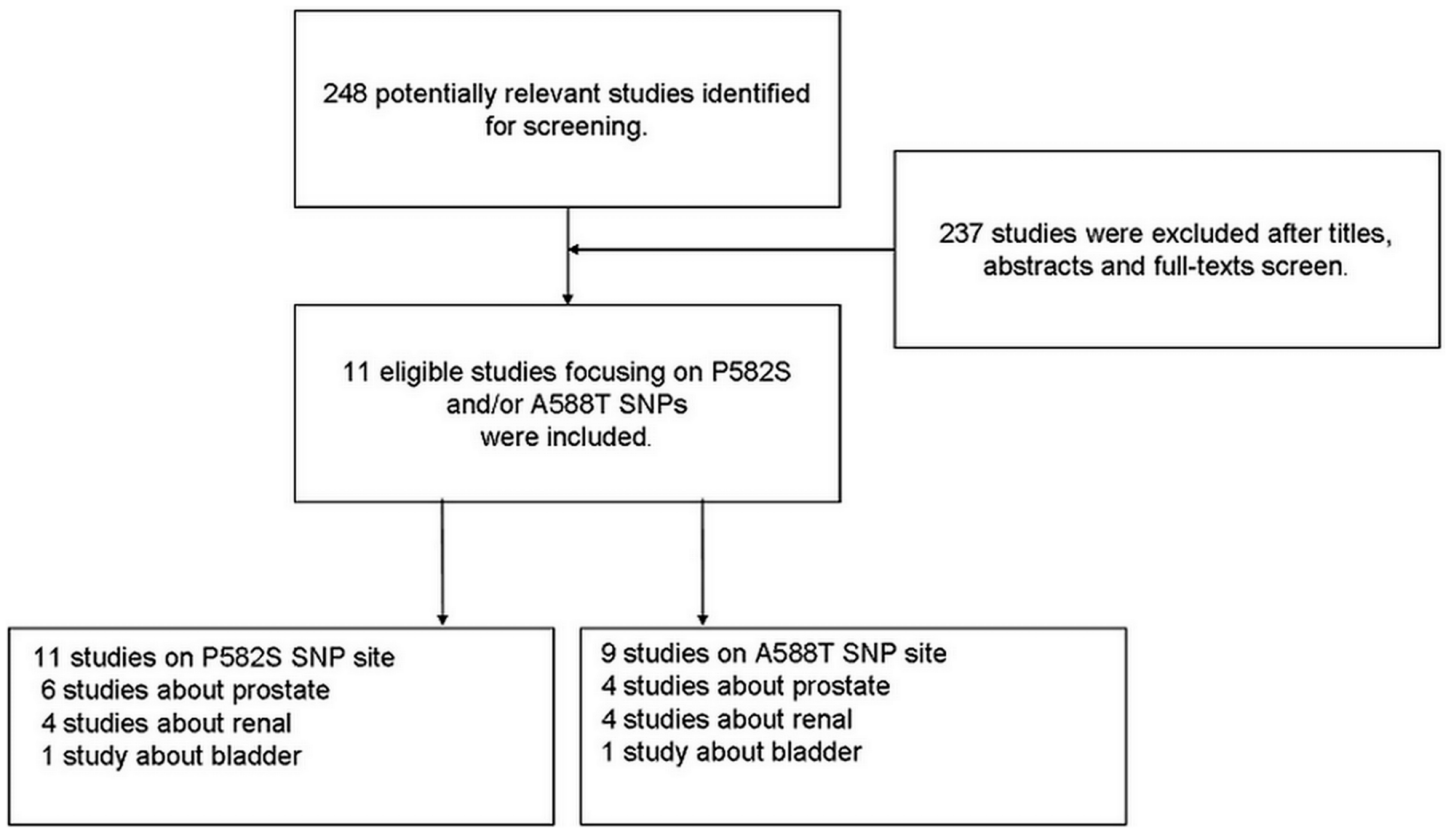
The flow diagram of search strategy in this meta-analysis.

Overall, a total of 248 abstracts meeting the search criteria were retrieved through PubMed. After screening titles, abstracts and full texts, we identified 11 qualified case-control studies exploring the relationship of HIF1A P582S and/or A588T polymorphisms and urinary cancers. The flow diagram of search strategy in this meta-analysis was shown in Fig. 1. 11 individual studies [11]–[21] with 5195 cases and 5786 controls for P582S polymorphism, and 9 studies [11]–[13], [15], [17]–[21] with 3482 cases and 4304 controls for A588T polymorphism were respectively included in the meta-analysis. Characteristics of included studies were summarized in Table 1. Three studies [12], [13], [17] included participants of Asian descent, five [11], [14], [15], [19], [21] included Caucasian and three [16], [18], [20] mixed population. Still, six studies [12], [14], [16], [18]–[20] only recruiting male subjects focused on prostate cancer, four [11], [13], [15], [21] with both male and female subjects on renal cell carcinoma, one study [17] with male and female participant on transitional cell carcinoma. In the study by Chau CH [20], there was no subject carrying mutant allele for the HIF1A A588T polymorphism. We decided to include this study based on consensus, and then did subgroup analysis by deleting the study.

**Table 1:** Characteristics of eligible studies included in the meta-analysis.

First Author	Year	Ref.	Country	Ethnicity	Gender	Cancer Types	SNPSites	Cases, n			Controls, n			Genotyping methods	HWE
								WW	WM	MM	WW	WM	MM		Y/N
Li P	2012	12	China	Asian	M	Prostate	P582S	612	48	2	659	57	0	Taqman	Y
							A588T	614	47	1	685	31	0	Taqman	Y
Qin C	2012	13	China	Asian	M/F	Renal	P582S	572	46	2	578	43	2	Taqman	Y
							A588T	575	45	0	584	39	0	Taqman	Y
Foley R	2009	14	Ireland	Caucasian	M	Prostate	P582S	65	30	0	175	13	0	Sequencing	Y
Morris MR	2009	15	Poland	Caucasian	M/F	Renal	P582S	290	39	3	262	46	5	Taqman	Y
							A588T	313	10	2	294	15	0	Taqman	Y
Jacobs EJ	2008	16	USA	Mixed	M	Prostate	P582S	1156	252	12	1138	284	28	Taqman	N
Nadaoka J	2008	17	Japan	Asian	M/F	Bladder	P582S	197	21	1	419	42	0	PCR-RFLP	Y
							A588T	204	13	2	421	40*		PCR-RFLP	Y
Orr-Urtreger A	2007	19	Israel	Caucasian	M	Prostate	P582S	287	99	16	217	80	3	PCR-RFLP	Y
							A588T	198	2	0	298	2	0	PCR-RFLP	Y
Li H	2007	18	USA	Mixed	M	Prostate	P582S	818	209	14	995	221	18	PCR-RFLP	Y
							A588T	1053	13	0	1247	17	0	PCR-RFLP	Y
Chau CH	2005	20	USA	Mixed	M	Prostate	P582S	161	29	6	179	14	3	Sequencing	N
							A588T	195	1	0	196	0	0	Sequencing	–
Ollerenshaw M	2004	21	UK	Caucasian	M/F	Renal	P582S	16	54	90	1	90	71	PCR-RFLP	N
							A588T	65	67	14	239	39	10	PCR-RFLP	N
Clifford SC	2001	11	UK	Caucasian	M/F	Renal	P582S	42	6	0	110	27	6	PCR-SSCP	N
							A588T	47	1	0	140	4	0	Sequencing	Y

W: wild type alleles (1772C or 1790G);

M: mutant type alleles (1772T or 1790A);

HWE: Hardy-Weinberg Equilibrium;

* Frequency of genotypes “AA+AG”.

### Frequency of HIF1A P582S and A588T Polymorphisms in Control Population

As for HIF1A P582S polymorphism, 1106 controls of Caucasian population and 1803 controls of Asian population were included in the meta-analysis. The frequencies of the C and T alleles for Caucasian were 80.74%, 19.26%, while those for Asian were 95.90% and 4.10%, respectively (Table 2). The frequencies of the CC, CT and TT genotypes for Caucasian were 69.17%, 23.15%, 7.69% respectively, while those for Asian were 91.90%, 7.99% and 0.11% (Table 2). The frequency distributions of the alleles and genotypes for HIF1A P582S polymorphism were obviously different between Caucasian and Asian groups (Table 2).

**Table 2:** The genotype and allele frequencies of HIF1A gene P582S and A588T polymorphisms in controls from Caucasian and Asian groups.

SNPs	Genotype/Allele		Caucasian		Asian		*P-*value
			n	%	n	%	
P582S	Genotypes	CC	765	69.17	1657	91.90	
		CT	256	23.15	144	7.99	
		TT	85	7.69	2	0.11	0.000^a^
		TT+CT	341	30.83	146	8.10	0.000^b^
	Alleles	C	1786	80.74	3458	95.90	
		T	426	19.26	148	4.10	0.000^c^
A588T	Genotypes	GG	971	93.28	1690	93.89	
		AA+AG	70	6.72	110	6.11	0.518^b^
	Alleles*	G	2002	96.16	2608	97.39	
		A	80	3.84	70	2.61	0.016^c^

* Study by Nadaoka J was not included;

a *P*<0.05 for the comparison between HIF1A gene P582S genotypes;

b *P* value for the dominant models;

c *P*<0.05 for the allele models.

As for HIF1A A588T polymorphism, The frequencies of the AA+AG and GG genotypes for Caucasian were 6.72%, 93.28% respectively, while those for Asian were 6.11% and 93.89%. The frequency distributions of the genotypes for HIF1A A588T polymorphism were statistically insignificant between the Caucasian and Asian groups. The frequencies of the A and G alleles for Caucasian were 3.84%, 96.16%, while those for Asian were 2.61% and 97.39%, respectively (Table 2). The frequency distributions of the alleles for HIF1A A588T polymorphism were obviously different between the Caucasian and Asian groups (Table 2).

### Main Results of Meta-analysis

**Figure 2:**
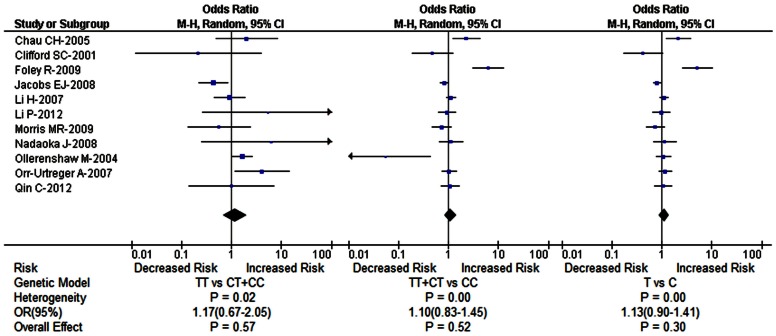
Forest plot of HIF1A gene P582S polymorphism and the risk of urinary cancers in the recessive, dominant and allele models.

The main results of meta-analysis about HIF1A P582S polymorphism were shown in Table 3. Firstly, we conducted meta-analysis of the effect of HIF1A P582S polymorphism on the susceptibility of urinary cancers based on 11 case-control studies (Table 3, Fig. 2). The results showed no significant association between the two in the dominant model (TT+CT vs CC OR = 1.10, 95% CI = 0.83–1.45, *P*_heterogeneity_ = 0.00, *P = *0.52), the recessive model (TT vs CT+CC OR = 1.17, 95% CI = 0.67–2.05, *P*_heterogeneity_ = 0.02, *P = *0.57) and the allele model (T vs C OR = 1.13, 95% CI = 0.90–1.41, *P*_heterogeneity_ = 0.00, *P = *0.30). Secondly, we performed subgroup meta-analysis based on the difference of ethnicity, cancer type and HWE status. We found that subjects with TT genotype had 1.60 fold higher risk than those with CC or CT genotype in Caucasian population (TT vs CT+CC OR = 1.60, 95% CI = 1.09–2.33, *P*_heterogeneity_ = 0.11, *P = *0.02). The remaining subgroup pooled ORs from this analysis were insignificant (all *P*>0.05) (Table 3).

**Table 3:** Main results of meta-analysis for the association of HIF1A gene P582S polymorphism and urinary cancers risk.

Genetic Model	Groups/Subgroups	Studies, n	Heterogeneity Test		Statistical Model	Test for Overall Effect		
			*I* ^2^, %	*P*		OR	95% CI	*P*
TT vs CT+CC	Overall	11	55	0.02	Random	1.17	0.67–2.05	0.57
	Overall in HWE	7	33	0.19	Fixed	1.38	0.85–2.26	0.19
	Caucasian	5	51	0.11	Fixed	1.60	1.09–2.33	**0.02**
	Caucasian in HWE	3	76	0.04	Random	1.57	0.22–11.14	0.65
	Asian	3	0	0.50	Fixed	2.38	0.60–9.39	0.22
	Prostate	6	69	0.01	Random	1.31	0.54–3.20	0.55
	Prostate in HWE	4	61	0.08	Random	2.03	0.58–7.16	0.27
	Renal	4	21	0.28	Fixed	1.37	0.92–2.04	0.12
	Renal in HWE	2	0	0.64	Fixed	0.69	0.22–2.17	0.52
TT+CT vs CC	Overall	11	80	0.00	Random	1.10	0.83–1.45	0.52
	Overall in HWE	7	77	0.00	Random	1.20	0.88–1.64	0.25
	Caucasian	5	89	0.00	Random	0.89	0.37–2.13	0.79
	Caucasian in HWE	3	92	0.00	Random	1.61	0.61–4.25	0.34
	Asian	3	0	0.86	Fixed	1.03	0.80–1.33	0.84
	Prostate	6	87	0.00	Random	1.36	0.95–1.96	0.09
	Prostate in HWE	4	87	0.00	Random	1.46	0.89–2.40	0.14
	Renal	4	70	0.02	Random	0.62	0.33–1.19	0.15
	Renal in HWE	2	29	0.23	Fixed	0.90	0.67–1.22	0.51
T vs C	Overall	11	78	0.00	Random	1.13	0.90–1.41	0.30
	Overall in HWE	7	75	0.00	Random	1.20	0.91–1.59	0.21
	Caucasian	5	86	0.00	Random	1.17	0.68–2.00	0.57
	Caucasian in HWE	3	92	0.00	Random	1.57	0.66–3.70	0.30
	Asian	3	0	0.88	Fixed	1.05	0.82–1.35	0.68
	Prostate	6	87	0.00	Random	1.35	0.96–1.89	0.08
	Prostate in HWE	4	85	0.00	Random	1.43	0.93–2.21	0.10
	Renal	4	44	0.15	Fixed	0.91	0.73–1.12	0.37
	Renal in HWE	2	37	0.21	Fixed	0.89	0.67–1.19	0.43

HWE: Hardy-Weinberg Equilibrium.

**Figure 3:**
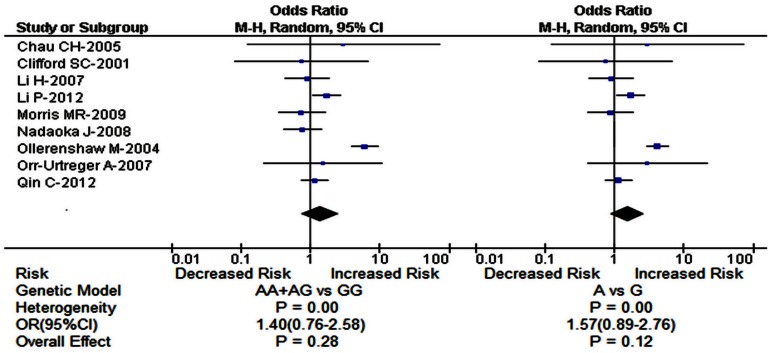
Forest plot of HIF1A gene A588T polymorphism and the risk of urinary cancers in the dominant and allele models.

The main results of meta-analysis about HIF1A A588T polymorphism were shown in Table 4. In the begin, we conducted meta-analysis of the effect of HIF1A A588T polymorphism on the susceptibility of urinary cancers based on 9 case-control studies (Table 4, Fig. 3). The results showed no significant association between the two in the dominant model (AA+AG vs GG OR = 1.40, 95% CI = 0.76–2.58, *P*_heterogeneity_ = 0.00, *P = *0.28) and the allele model (A vs G OR = 1.57, 95% CI = 0.89–2.76, *P*_heterogeneity_ = 0.00, *P = *0.12). Subsequently, we performed subgroup meta-analysis based on the difference of ethnicity, cancer type and HWE status. We found that subjects carrying A allele had 1.45 fold higher risk than those with GG genotype in prostate cancer (AA+AG vs GG OR = 1.45, 95% CI = 1.00–2.12, *P*_heterogeneity_ = 0.50, *P = *0.05). Still, significant associations were found in the allele model in prostate cancer (A vs G OR = 1.41, 95% CI = 1.03–1.93, *P*_heterogeneity_ = 0.22, *P = *0.03), prostate cancer in HWE (A vs G OR = 1.45, 95% CI = 1.00–2.11, *P*_heterogeneity_ = 0.33, *P = *0.05) and Asian population (A vs G OR = 1.46, 95% CI = 1.01–2.12, *P*_heterogeneity_ = 0.49, *P = *0.04). The remaining subgroup pooled ORs from this analysis were insignificant (all *P*>0.05) (Table 4).

**Table 4:** Main results of meta-analysis for the association of HIF1A gene A588T polymorphism and urinary cancers risk.

Genetic Model	Groups/Subgroups	Studies, n	Heterogeneity Test		Statistical Model	Test for Overall Effect		
			*I* ^2^, %	*P*		OR	95% CI	*P*
AA+AG vs GG	Overall	9	83	0.00	Random	1.40	0.76–2.58	0.28
	Overall in HWE	7	5	0.39	Fixed	1.13	0.89–1.44	0.32
	Caucasian	4	87	0.00	Random	1.67	0.39–7.07	0.49
	Caucasian in HWE	3	0	0.81	Fixed	0.82	0.41–1.62	0.56
	Asian	3	53	0.12	Fixed	1.24	0.94–1.64	0.14
	Prostate	4	0	0.50	Fixed	1.45	1.00–2.12	**0.05**
	Prostate in HWE	3	7	0.34	Fixed	1.44	0.98–2.10	0.06
	Renal	4	92	0.00	Random	1.58	0.49–5.03	0.44
	Renal in HWE	3	0	0.59	Fixed	1.04	0.71–1.51	0.85
A vs G	Overall	8	79	0.00	Random	1.57	0.89–2.76	0.12
	Overall in HWE	6	0	0.56	Fixed	1.24	0.96–1.62	0.10
	Caucasian	4	81	0.00	Random	1.64	0.53–5.10	0.39
	Caucasian in HWE	3	0	0.87	Fixed	0.92	0.48–1.78	0.81
	Asian	2	35	0.22	Fixed	1.41	1.03–1.93	**0.03**
	Prostate	4	0	0.49	Fixed	1.46	1.01–2.12	**0.04**
	Prostate in HWE	3	10	0.33	Fixed	1.45	1.00–2.11	**0.05**
	Renal	4	89	0.00	Random	1.53	0.60–3.92	0.38
	Renal in HWE	3	0	0.78	Fixed	1.07	0.74–1.55	0.71

HWE: Hardy-Weinberg Equilibrium.

### Heterogeneity, Sensitivity and Publication Bias Tests

Significant heterogeneity was observed in some comparisons (*P*<0.10), and results were listed in Table 3 and 4. Sensitivity analysis was carried out by performing subgroup analysis of studies with controls in HWE (Table 3 and 4). The result of the recessive model comparison showed no evidence that HIF1A P582S polymorphism conferred to an increased urinary cancers risk in Caucasian population (TT vs CT+CC OR = 1.57, 95% CI = 0.22–11.14, *P*_heterogeneity_ = 0.04, *P = *0.65) (Table 3). The other results of subgroup analysis showed no difference between including and excluding studies with controls not in HWE.

**Figure 4:**
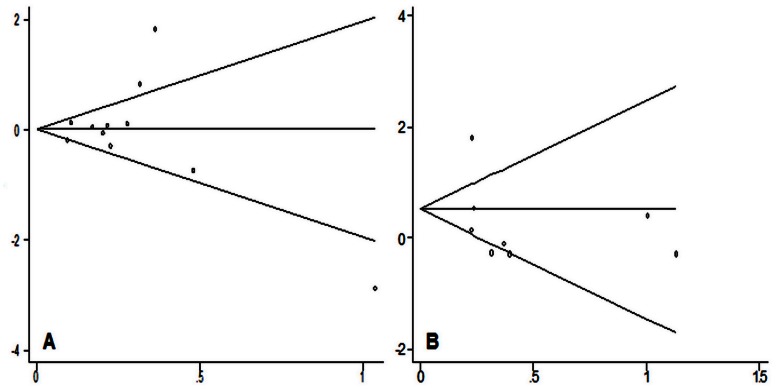
Results of Begg’s test for HIF1A gene C1772T (A) and G1790A (B) polymorphisms in the dominant model.

The potential publication bias was firstly appraised by the funnel plot which showed no apparently asymmetric. Still, the results of Begg’s test revealed no publication bias (*P*>0.05). The results of Begg’s test in the dominant model for HIF1A P582S and A588T polymorphisms were shown in Fig. 4.

## Discussion

Hypoxia is one of the fundamentally important features of solid tumors. Cellular response to hypoxia is partially governed by the activation of HIF1, which functions as a global regulator of oxygen homeostasis. HIF1 is a dimeric protein complex of α and β subunits, both of which are members of the basic helix-loop-helix Per/Arnt/Sim transcription factor family [25]. HIF1A contains several functional elements, including bHLH, PAS, N-TAD, C-TAD and ODD. Under normoxia, HIF1A is hydroxylated on proline residues 402 and 564 within the HIF1A ODD in the presence of iron [26] by oxygen-dependant prolyl hydroxylases. Then an E3 ubiquitin ligase, the von Hippel-Lindau tumor suppressor protein (pVHL), targets hydroxylated HIF1A for rapid degradation [27]. In circumstance with low oxygen concentration, the described protein degradation pathway was shut down. Cumulative HIF1A plays its regulatory role in the hypoxic response and adaption pathway through regulating more than sixty downstream molecules via cognate hypoxia response elements in their promoters. Still, the proximity of these polymorphisms near N-TAD may affect conformation and function. Thereby, changes of amino acids near the N-TAD of HIF1A have the possibility to change its transactivation activity. One well-known pathway is activating angiogenesis to combat hypoxia. Accumulating HIF1A translocates into the nucleus and activates the expression of the most prominent target gene, vascular endothelial-derived growth factor (VEGF) [5]. Another pathway is that HIF1A downregulates functions of DNA repair genes. Recent studies demonstrated that HIF1A inhibited the DNA mismatch repair system, such as MLH1, MSH2 and MSH6 [28], [29]. Thus, genomic instability will have a higher possibility of activation of oncogenes to promote tumor progression. Still, HIF1A can protect tumor cells from hypoxia to survive and grow by the means of promoting proliferation, becoming resistant to apoptosis, switching to a glycolytic metabolism, evading immune attack, migrating to less hypoxia areas of the body, and so on [30]. Therefore, factors whichever influence the quantity and/or activity of HIF-1A will definitely affect the onset and fate of tumor cells.

Genetic differences are partly responsible for inter-individual diversity and variation in the development of complex diseases. SNP is one of the common genetic alterations, which serves as a new method for screening the etiology of cancer with complex inheritance [31]–[33]. HIF1A, the main regulatory subunit of HIF-1, harbors hundreds of polymorphism sites. Recently, HIF1A gene polymorphisms have been evaluated for a probable role in mediating genetic predisposition to cancer. Two missense polymorphisms, HIF1A P582S and A588T, were most widely studied SNP sites, which were then supposed to modify the risk of urinary cancers, such as renal, urothelial and prostate cancers. However, the main results of single case-control studies finally yield inconsistent conclusions. In this study, we aimed to conduct a comprehensive meta-analysis to get a clear association between the two.

HIF1A A588T, also termed as Ala588Thr, G1790A, rs11549467, is located within the oxygen-dependent degradation domain (ODD) which spans from amino acid 401 to 603. In normoxia, HIF1A is hydroxylated on Pro402 and Pro564 followed by interaction with VHL to initiate rapid ubiquitination and proteasomal degradation. This may be one of the precise mechanisms that HIF1A A588T polymorphism plays its effect. In our study, the A allele was significantly correlated with higher urinary cancers risk in Asian population (OR = 1.41, 95% CI = 1.03–1.93, *P*_heterogeneity_ = 0.22, *P* = 0.03). Still, a significant association was found for prostate cancer in allele model (OR = 1.46, 95% CI = 1.01–2.12, *P*_heterogeneity_ = 0.49, *P* = 0.04). A marginal significant association between the two was detected for prostate cancer in the dominant model by analyzing only studies with controls in HWE (OR = 1.45, 95% CI = 1.00–2.12, *P*_heterogeneity_ = 0.50, *P* = 0.05). The remaining pooled ORs from this analysis were insignificant (all *P*>0.05). Studies for mechanism found that A588T variant showed a higher transactivation capacity than WT under either normoxic or hypoxic condition [34]. The same group also provided evidence to support their in vitro results with in vivo studies that tumors with rare allele had significantly higher number of microvessels [34].

HIF1A P582S, also termed as Pro582Ser, C1772T, rs11549465, is located in exon 12 near Pro564 within the ODD, which is supposed to affect the hydroxylation of Pro564 as HIF1A A588T. Additionally, this position is also located near the N-terminal transactivation domain (TAD-N), which spans from amino acid 531 to 575. Transcriptional activity of HIF-1 is facilitated by TAD-N and TAD-C in HIF1A and one another in HIF1B. In our study, individuals with TT genotype showed 1.60 fold higher risk than the other carrying CT or CC genotypes in Caucasian population (OR = 1.60, 95% CI = 1.09–2.33, *P*_heterogeneity_ = 0.11, *P* = 0.02). The remaining pooled ORs from this analysis were insignificant (all *P*>0.05). Studies for mechanism found that P582S variant showed a higher transactivation capacity than WT under either normoxic or hypoxic condition [34], [35], which was tested in vivo samples in the same study [34]. Still, evidence suggested that P582S mutation which blocked proline hydroxylation dependent degradation showed increased protein stability under normoxia [35]. However, the sensitivity analysis by deleting studies with controls deviating from HWE did not show a significant association (OR = 1.45, 95% CI = 1.00–2.11, *P*_heterogeneity_ = 0.33, *P* = 0.65). Because the results of the sensitivity analysis excluding studies not in HWE would be more reliable [36], our previous conclusion that HIF1A P582S confers susceptibility to urinary cancers should be validated with future studies.

The current evidences suggest that HIF1A P582S polymorphism may correlate with urinary cancers risk in Caucasian population, while HIF1A A588T polymorphism increases the risk of urinary cancers in Asian population. Ethnicity may be an essential biological factor which influences HIF1A P582S and/or A588T polymorphisms through gene-gene interactions. As we presented in Table 2, the genotype and allele frequencies of these two SNPs were apparently different among controls recruited in our study (*P*<0.05). As for HIF1A P582S polymorphism, 1106 controls of Caucasian and 1803 controls of Asian population were included in the meta-analysis. The frequencies of the T, CT, TT and CT+TT for Caucasian were 19.26%, 23.15%, 7.69%, and 30.83% respectively, higher than those for Asian population 4.10%, 7.99%, 0.11%, and 8.1%. As for HIF1A A588T polymorphism, 1041 controls of Caucasian and 1800 controls of Asian population were included in the meta-analysis. The frequencies of A and AA+AG for Caucasian were 3.84%, 6.72% respectively, while those for Asian were 2.61% and 6.11%. The frequency distributions of the alleles for HIF1A A588T polymorphism were statistically significant between the Caucasian and Asian groups.

Some studies reported that HIF1A P582S and A588T polymorphisms increased the risk of urinary cancers, while others failed to replicate the association between the two. The inconsistent results may largely derive from small sample size, different designed methods and complex genetic backgrounds. In our study, we conducted meta-analysis to get conclusions of higher statistical power. To our best knowledge, this is the first meta-analysis evaluating the association between HIF1A P582S and A588T polymorphisms and the susceptibility of urinary cancers. On the other hand, there were some limitations similar to other meta-analyses which might affected the final results of our study. We performed a systematic search to find as complete published case-control studies as possible. However, a few studies would not have been included in the meta-analysis. Also, the number of eligible studies as well as included cases and controls for some analyses was not large enough. Thereby, we were actually underpowered to get significant associations. Moreover, our final results were based on unadjusted estimates. A more precise analysis stratified by age, different gender, lifestyle, and stages/grades of cancers should be conducted as individual studies were available.

In the present study, we provide preliminarily genetic evidence that HIF1A P582S polymorphism is a potential factor for the susceptibility of urinary cancers in Caucasian population, while A588T polymorphism contributes to the risk of urinary cancers in Asian population and prostate cancer. Due to existing limitations, our conclusions should be interpreted with caution. Additional well-designed studies with larger sample size focusing on gene-gene and gene-environment are required to present robust evidence for the associations. Still, further molecular studies are warranted to clarify the effects of HIF1A P582S and A588T polymorphisms on the onset and progression of urinary cancers.
